# Imaging of Tumor Stroma Using ^68^Ga-FAPI PET/CT to Improve Diagnostic Accuracy of Primary Tumors in Head and Neck Cancer of Unknown Primary: A Comparative Imaging Trial

**DOI:** 10.2967/jnumed.123.266556

**Published:** 2024-03

**Authors:** Bingxin Gu, Ziyi Yang, Xinyue Du, Xiaoping Xu, Xiaomin Ou, Zuguang Xia, Qing Guan, Silong Hu, Zhongyi Yang, Shaoli Song

**Affiliations:** 1 Department of Nuclear Medicine, Fudan University Shanghai Cancer Center, Shanghai, China;; 2 Department of Oncology, Shanghai Medical College, Fudan University, Shanghai, China;; 3 Center for Biomedical Imaging, Fudan University, Shanghai, China;; 4 Shanghai Engineering Research Center of Molecular Imaging Probes, Shanghai, China;; 5 Department of Radiation Oncology, Fudan University Shanghai Cancer Center, Shanghai, China;; 6 Department of Medical Oncology, Fudan University Shanghai Cancer Center, Shanghai, China; and; 7 Department of Head and Neck Surgery, Fudan University Shanghai Cancer Center, Shanghai, China

**Keywords:** fibroblast activation protein, FDG, PET/CT, head and neck cancer, cancer of unknown primary

## Abstract

The low detection rate of primary tumors by current diagnostic techniques remains a major concern for patients with head and neck cancer of unknown primary (HNCUP). Therefore, in this study, we aimed to investigate the potential role of ^68^Ga-labeled fibroblast activation protein inhibitor (^68^Ga-FAPI) PET/CT compared with ^18^F-FDG PET/CT for the detection of primary tumors of HNCUP. **Methods:** In this prospective comparative imaging trial conducted at Fudan University Shanghai Cancer Center, 91 patients with negative or equivocal findings of a primary tumor by comprehensive clinical examination and conventional imaging were enrolled from June 2020 to September 2022. The presence of a primary tumor was recorded by 3 experienced nuclear medicine physicians. Primary lesions were validated by histopathologic analysis and a composite reference standard. **Results:** Of the 91 patients (18 women, 73 men; median age, 60 y; age range, 24–76 y), primary tumors were detected in 46 (51%) patients after a thorough diagnostic work-up. ^68^Ga-FAPI PET/CT detected more primary lesions than ^18^F-FDG PET/CT (46 vs. 17, *P* < 0.001) and showed better sensitivity, positive predictive value, and accuracy in locating primary tumors (51% vs. 25%, 98% vs. 43%, and 51% vs. 19%, respectively). Furthermore, ^68^Ga-FAPI PET/CT led to treatment changes in 22 of 91 (24%) patients compared with ^18^F-FDG PET/CT. The Kaplan–Meier curve illustrated that patients with unidentified primary tumors had a significantly worse prognosis than patients with identified primary tumors (hazard ratio, 5.77; 95% CI, 1.86–17.94; *P* = 0.0097). **Conclusion:** ^68^Ga-FAPI PET/CT outperforms ^18^F-FDG PET/CT in detecting primary lesions and could serve as a sensitive, reliable, and reproducible imaging modality for HNCUP patients.

## Visual Abstract

**Figure:**
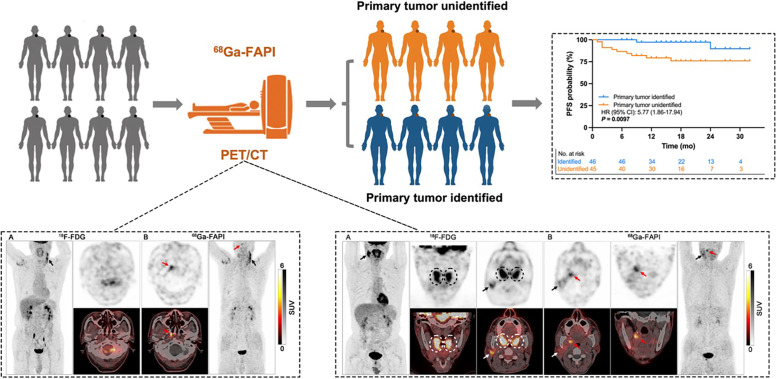

Head and neck cancer of unknown primary (HNCUP) is a group of highly heterogeneous malignancies and usually manifests as an enlarged cervical lymph node at initial diagnosis (1). The low incidence of HNCUP, accounting for 1%–5% of all head and neck cancers (2), and the uneven medical level lead to a lack of normative experience among different medical centers in locating primary tumors. The increase in multidisciplinary teams may improve the quality of assessment and management for HNCUP patients based on previously proposed guidelines, for example, the National Comprehensive Cancer Network (3) and American Society of Clinical Oncology (4) guidelines. Nevertheless, the low detection rate of primary tumors by current diagnostic techniques (e.g., CT, MRI, nasopharyngoscopy, and laryngoscopy) remains a major concern for patients with HNCUP (5).

Molecular imaging using ^18^F-FDG PET/CT improves the detection of primary tumors compared with CT and MRI by reflecting the level of glucose metabolism in tumor cells (6). Schaarschmidt et al. (7) demonstrated that ^18^F-FDG PET/CT or PET/MRI outperformed MRI alone for T staging in terms of accuracy (59% or 75% vs. 50%). However, elevated nonspecific uptake of ^18^F-FDG by normal tissues or inflammatory cells in the head and neck region may lead to false-positive findings and may conceal small primary tumors, especially in the oropharynx, resulting in a false-negative diagnosis (8–10). In addition to noninvasive and minimally invasive methods, diagnostic tonsillectomy is recommended for patients with metastatic squamous cell carcinoma on the neck and human papillomavirus positivity but no obvious signs of primary tumors on clinical examination, imaging, or panendoscopy (4). Alzahrani et al. (11) reported a detection rate of 49.2% for locating primary tumors via transoral robotic mucosectomy in 65 patients with negative findings on comprehensive clinical examination and standard imaging. Nevertheless, postoperative complications, for example, pneumonia, feeding difficulty, and hemorrhage, may prolong hospitalization and delay antineoplastic therapy (12). Therefore, noninvasive diagnostic techniques to improve the detection of primary tumors before definitive therapy for HNCUP patients are urgently need.

Recently, PET imaging targeting fibroblast activation protein (FAP) has shown great potential in depicting non–^18^F-FDG-avid malignant tumors (13,14). FAP is overexpressed on cancer-associated fibroblasts, which account for most tumor stromata in more than 90% of epithelial carcinomas (15). By imaging the tumor stroma rather than tumor cells, ^68^Ga-labeled FAP inhibitor (FAPI) PET/CT reveals elevated radioactivity on primary and metastatic lesions and low background uptake in normal tissues among various tumors, including gastrointestinal tumors (16), hepatobiliary tumors (17), and head and neck cancers (18). Serfling et al. (18) demonstrated that noninvasive imaging of FAP expression by ^68^Ga-FAPI PET/CT resulted in better visual detection of the malignant primary tumors in the Waldeyer tonsillar ring, thereby avoiding diagnostic tonsillectomy.

Inspired by the promising results of ^68^Ga-FAPI PET/CT imaging in patients with various head and neck cancers (e.g., nasopharyngeal carcinoma, oropharyngeal cancer, and salivary ductal carcinoma), we hypothesized that ^68^Ga-FAPI PET/CT would outperform ^18^F-FDG PET/CT in localizing primary tumors in HNCUP patients. Thus, in this study, we aimed to investigate the potential usefulness of ^68^Ga-FAPI PET/CT compared with ^18^F-FDG PET/CT for the detection of primary tumors in patients with HNCUP. The primary objective of this study was to compare the sensitivity, positive predictive value, and accuracy of ^68^Ga-FAPI and ^18^F-FDG PET/CT in localizing primary tumors. Secondary objectives were to compare ^68^Ga-FAPI and ^18^F-FDG uptake by primary and metastatic lesions and progression-free survival.

## MATERIALS AND METHODS

### Patients

This was a prospective comparative imaging trial performed at Fudan University Shanghai Cancer Center from June 2020 to September 2022. Patients were eligible if they met the following inclusion criteria: older than 18 y, pathology-confirmed metastatic cervical carcinoma, negative or equivocal finding of a primary tumor by comprehensive clinical examination and conventional imaging modalities (e.g., contrast-enhanced CT and MRI), and paired ^18^F-FDG and ^68^Ga-FAPI PET/CT scans within 1 wk. Patients with non–head and neck primary carcinomas, lymphoepitheliomalike carcinoma, 2 or more malignances, and unavailable clinical data were excluded.

The Standards for Reporting of Diagnostic Accuracy checklist is included in Supplemental Data 1 (supplemental materials are available at http://jnm.snmjournals.org), and the flow diagram is shown in Supplemental Figure 1 and Supplemental Data 2. The study was approved by the Fudan University Shanghai Cancer Center Institutional Review Board (2004216-25), and written informed consent was obtained from each patient. The data of 18 patients have been reported previously (10).

### PET/CT Acquisition and Image Interpretation

^18^F-FDG and ^68^Ga-FAPI PET/CT were performed within 1 wk. The 2 radionuclide PET/CT scans were obtained from a Biograph mCT Flow scanner (Siemens Medical Solutions). The detailed protocols for image acquisition and reconstruction are presented in Supplemental Data 2 (19).

Three experienced nuclear medicine physicians analyzed and interpreted the ^18^F-FDG and ^68^Ga-FAPI PET/CT images independently, and they reached a consensus in cases of inconsistency. Lesions with increased radioactivity compared with the surrounding normal tissue and not associated with physiologic uptake were considered suspected malignant lesions. SUV_max_ and SUV_mean_ normalized to body weight were manually computed by drawing a 3-dimensional volume of interest for the tumor lesion and normal liver, respectively. Meanwhile, the tumor-to-liver ratio (TLR) was calculated according to the following formula: TLR = tSUV_max_/lSUV_mean_, where tSUV_max_ is the SUV_max_ of the tumor lesion and lSUV_mean_ is the SUV_mean_ of the liver.

### Clinical Assessment and Follow-up

All suspected primary sites detected by ^18^F-FDG or ^68^Ga-FAPI PET/CT were verified by biopsy or histopathologic examination. Suspected metastatic lesions were confirmed by biopsy or 6-mo follow-up. Suspected metastatic lesions with typical malignant features on PET/CT images or a significant reduction or progression in size after anticancer treatment during follow-up were considered malignant. After a thorough diagnostic work-up, including medical history, imaging, and endoscopy or tonsillectomy, all patients with or without an identified primary tumor received treatment based on the decision of the multidisciplinary head and neck cancer team. Treatment response was assessed by imaging examination according to RECIST version 1.1 (20). The endpoint was set as progression-free survival, defined as the time randomization to disease progression or death.

### Statistical Analysis

Differences in general information between patients with identified and those with unidentified primary tumors were evaluated using the Mann–Whitney test (for continuous characteristics) and the χ^2^ test or Fisher exact test (for discrete characteristics). The differences in SUV_max_ and TLR between ^18^F-FDG and ^68^Ga-FAPI PET/CT were assessed using the paired *t* test and Wilcoxon signed-rank test, respectively. Diagnostic performance was evaluated by receiver-operating-characteristic curve analysis. The survival analyses were performed using the Kaplan–Meier method. SPSS version 26 (IBM) was used for statistical analyses. A 2-tailed *P* value of less than 0.05 was considered statistically significant.

## RESULTS

### Patient Characteristics

From June 2020 to September 2022, 91 patients (18 women, 73 men; median age, 60 y; age range, 24–76 y) were enrolled in this prospective study. Of the 91 patients, a primary tumor was detected in 46 (51%) patients after a thorough diagnostic work-up. The baseline characteristics for the patients with identified and unidentified primary tumors are presented in Table 1 and Supplemental Data 3. Among these clinical characteristics, the presence of Epstein–Barr virus DNA and the Epstein–Barr virus–encoded small RNA status showed significant differences between these 2 cohorts, whereas there was no significant difference in the human papillomavirus or p16 status. With regard to the therapeutic regimen, chemotherapy and radiotherapy were the main choices for patients with identified primary tumors, whereas chemotherapy was the main choice for patients with unidentified primary tumors.

**TABLE 1.** Baseline Characteristics

Characteristic	Total, *n* = 91	Primary tumor identified, *n* = 46	Primary tumor unidentified, *n* = 45	*P*
Sex				0.793
Female	18 (20)	10 (22)	8 (18)	
Male	73 (80)	36 (78)	37 (82)	
Age (y)	60 (24–76)	55 (33–76)	61 (24–73)	0.238
Body mass index (kg/m^2^)	23 (10–31)	23 (10–29)	24 (18–31)	0.149
Pathologic type of cervical lymph node				0.198
Squamous cell carcinoma	81 (89)	42 (91)	39 (86)	
Adenocarcinoma	7 (8)	4 (9)	3 (7)	
Poorly differentiated carcinoma	3 (3)	0 (0)	3 (7)	
EBV DNA status				0.024*
Positive	16 (17)	13 (28)	3 (7)	
Negative	47 (52)	20 (44)	27 (60)	
Unknown	28 (31)	13 (28)	15 (33)	
Human papillomavirus status				0.787
Positive	10 (11)	6 (13)	4 (9)	
Negative	11 (12)	5 (11)	6 (13)	
Unknown	70 (77)	35 (76)	35 (78)	
EBV-encoded RNA status				0.028*
Positive	18 (20)	13 (28)	5 (11)	
Negative	39 (43)	14 (31)	25 (56)	
Unknown	34 (37)	19 (41)	15 (33)	
p16 status				0.405
Positive	20 (22)	12 (26)	8 (18)	
Negative	27 (30)	11 (24)	16 (35)	
Unknown	44 (48)	23 (50)	21 (47)	
Surgery				0.677
Yes	45 (49)	24 (52)	21 (47)	
No	46 (51)	22 (48)	24 (53)	
Chemotherapy				0.026*
Yes	70 (77)	40 (87)	30 (67)	
No	21 (23)	6 (13)	15 (33)	
Radiotherapy				<0.001*
Yes	54 (59)	37 (80)	17 (38)	
No	37 (41)	9 (20)	28 (62)	
Targeted therapy				0.231
Yes	12 (13)	4 (9)	8 (18)	
No	79 (87)	42 (91)	37 (82)	
Immunotherapy				0.714
Yes	8 (9)	5 (11)	3 (7)	
No	83 (91)	41 (89)	42 (93)	
Progression-free survival				0.014*
Progression	12 (13)	2 (4)	10 (22)	
Progression-free	79 (87)	44 (96)	35 (78)	
Follow-up (mo)	19 (7–33)	18 (7–32)	19 (7–33)	0.708

* Statistically significant at *P* < 0.05.

EBV = Epstein–Barr virus.

Qualitative data are number and percentage; continuous data are median or mean and range.

### Assessment of Metastatic Lesions on ^18^F-FDG and ^68^Ga-FAPI PET/CT

**FIGURE 1.**
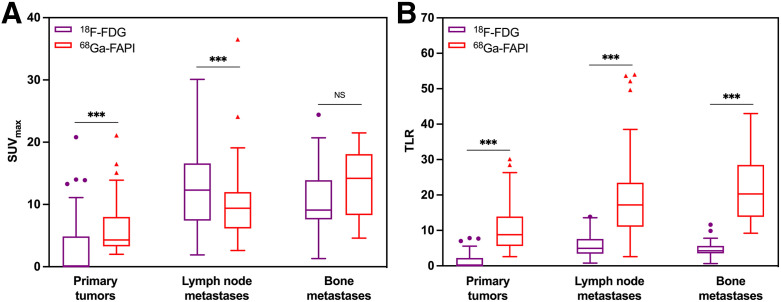
Box plots of SUV_max_ (A) and TLR (B) detected on ^18^F-FDG and ^68^Ga-FAPI PET/CT. Primary tumors showed significantly higher semiquantitative uptake of ^68^Ga-FAPI than ^18^F-FDG (*P* < 0.001). ^18^F-FDG outperformed ^68^Ga-FAPI PET/CT in detecting lymph node metastases, with significantly higher SUV_max_ (*P* < 0.001). In terms of TLR, lymph node and bone metastases presented more favorable uptake of ^68^Ga-FAPI than ^18^F-FDG (*P* < 0.001). ***P < 0.001. NS = no significance.

In total, 121 lymph node metastases and 15 bone metastases were involved in the analysis (Fig. 1; Supplemental Table 1). In terms of lymph node metastases, ^18^F-FDG PET/CT detected all metastatic lesions with significantly higher semiquantitative SUV_max_ than ^68^Ga-FAPI PET/CT (12.48 ± 6.10 and 9.80 ± 5.02, respectively; *P* < 0.001). Nevertheless, TLR presented more favorable uptake of ^68^Ga-FAPI than ^18^F-FDG (18.65 ± 10.50 and 5.64 ± 2.81, respectively; *P* < 0.001). With regard to bone metastases, ^68^Ga-FAPI PET/CT outperformed ^18^F-FDG PET/CT in terms of SUV_max_ (13.65 ± 5.12 and 10.85 ± 6.17, respectively; *P* = 0.173) and TLR (21.99 ± 9.70 and 4.94 ± 2.90, respectively; *P* < 0.001).

### Evaluation of Primary Tumors on ^18^F-FDG and ^68^Ga-FAPI PET/CT

**FIGURE 2.**
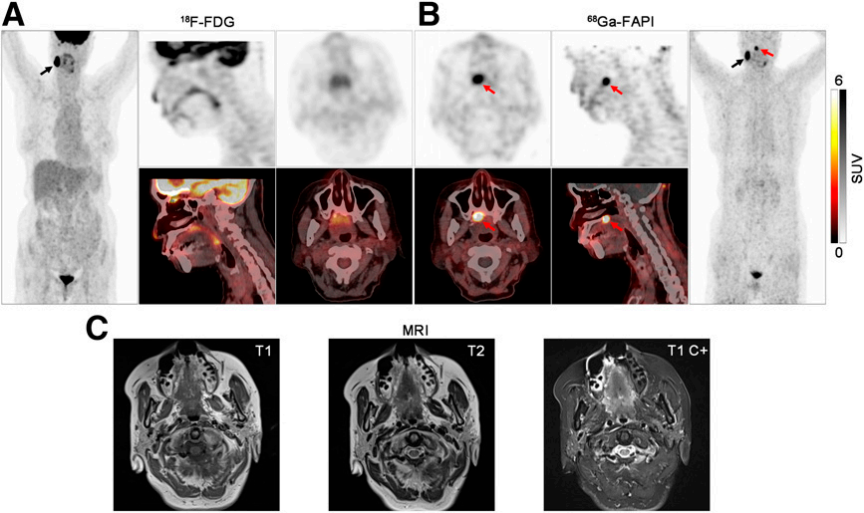
PET/CT and MR images of 72-y-old woman (patient 30) pathologically confirmed with metastatic squamous cell carcinoma of right neck. (A) ^18^F-FDG PET images (left and top) and PET/CT images (bottom), shown in coronal, sagittal, and axial views (from left to right), demonstrated metastatic lymph node of right neck with intensive metabolic activity (black arrow, SUV_max_, 30.1) but presented no evidence for primary tumor. (B) ^68^Ga-FAPI PET images (top and right) and PET/CT images (bottom), shown in axial, sagittal, and coronal views (from left to right), also detected metastatic lymph node with high ^68^Ga-FAPI activity (black arrow, SUV_max_, 16.3). There was intensive uptake of ^68^Ga-FAPI in palate (red arrow, SUV_max_, 11.3). (C) T1-weighted, T2-weighted, and contrast-enhanced T1-weighted MRI also presented no evidence for primary tumor. Subsequent surgery confirmed mucoepidermoid carcinoma of palate. C+ = contrast-enhanced.

Among the 46 patients with identified primary tumors, 39 patients received confirmation by pathology, whereas the other 7 patients were pathologically negative but diagnosed clinically. Table 2 shows that the locations of primary tumors included the nasopharynx (*n* = 14; Supplemental Fig. 2), tonsil (*n* = 21; Supplemental Fig. 3), submandibular gland (*n* = 3), thyroid (*n* = 3), hypopharynx (*n* = 2), tongue (*n* = 1), laryngopharynx (*n* = 1), and palate (*n* = 1; Fig. 2; Supplemental Fig. 4). Among the 7 patients with the primary tumor diagnosed clinically, 4 patients were diagnosed as having nasopharyngeal carcinoma with metastatic cervical nonkeratinizing squamous cell carcinoma and Epstein–Barr virus infection, and the other 3 patients were diagnosed as having tonsil carcinoma with metastatic cervical squamous cell carcinoma and moderate to severe dysplasia of tonsil squamous epithelial cells.

**TABLE 2.** Comparison of Primary Tumors Detected on ^18^F-FDG and ^68^Ga-FAPI PET/CT

Primary tumor site	Total (*n*)	^18^F-FDG	^68^Ga-FAPI	Treatment change led by ^68^Ga-FAPI
Nasopharynx	14 (4)	6	14 (4)	4
Tonsil	21 (3)	5	21 (3)	13
Palatine tonsil	13 (2)	3	13 (2)	8
Lingual tonsil	8 (1)	2	8 (1)	5
Submandibular gland	3	1	3	2
Thyroid	3	3	3	0
Hypopharynx	2	0	2	2
Tongue	1	1	1	0
Laryngopharynx	1	1	1	0
Palate	1	0	1	1
Total, *n* = 91	46 (51%)	17 (19%)	46 (51%)	22 (24%)

Primary tumor site data in parentheses indicate primary tumor was pathologically negative but diagnosed clinically. Total tumor data are number and percentage (*P* < 0.001).

Primary tumors in 17 of 91 (19%) patients were identified by ^18^F-FDG PET/CT. ^68^Ga-FAPI PET/CT showed a significantly higher detection rate (51%) of primary tumors than did ^18^F-FDG PET/CT (*P* < 0.001). Furthermore, ^68^Ga-FAPI PET/CT led to treatment changes in 22 of 91 (24%) patients compared with ^18^F-FDG PET/CT. Moreover, in terms of SUV_max_ and TLR, primary tumors demonstrated significantly higher semiquantitative uptake of ^68^Ga-FAPI than ^18^F-FDG (SUV_max_, 6.11 ± 4.30 and 3.16 ± 5.11, *P* < 0.001; TLR, 10.85 ± 6.81 and 1.45 ± 2.31, *P* < 0.001).

With regard to diagnostic performance in identifying primary tumors, contrast-enhanced MRI and ^18^F-FDG PET/CT showed similar sensitivity, positive predictive value, and accuracy, whereas contrast-enhanced CT showed the lowest sensitivity and accuracy (Table 3). ^68^Ga-FAPI PET/CT outperformed contrasted-enhanced CT, contrast-enhanced MRI, and ^18^F-FDG PET/CT in terms of sensitivity (51% vs. 17%, 27%, and 25%, respectively), positive predictive value (98% vs. 44%, 42%, and 43%, respectively), and accuracy (51% vs. 14%, 20%, and 19%, respectively).

**TABLE 3.** Diagnostic Performance of Contrast-Enhanced CT, Contrast-Enhanced MRI, ^18^F-FDG, and ^68^Ga-FAPI PET/CT in Identifying Primary Tumors

Test characteristic	Contrast-enhanced CT	Contrast-enhanced MRI	^18^F-FDG	^68^Ga-FAPI
True-positive (*n*)	12	15	17	46
False-positive (*n*)	15	21	23	1
False-negative (*n*)	60	40	51	44
Sensitivity (%)	17	27	25	51
Positive predictive value (%)	44	42	43	98
Accuracy rate (%)	14	20	19	51

### Survival Outcome

**FIGURE 3.**
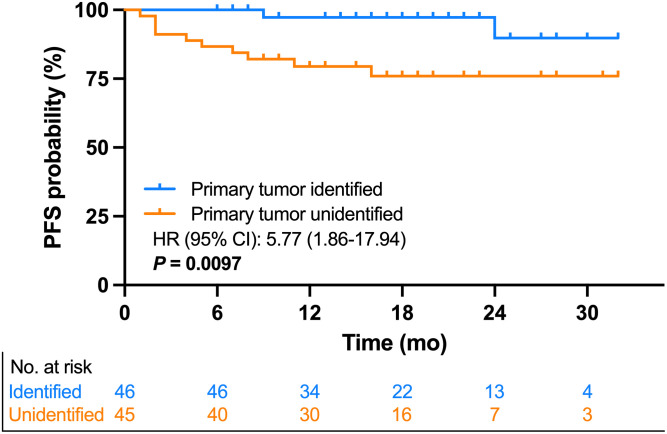
Kaplan–Meier curve for progression-free survival (PFS). HR = hazard rate.

After PET/CT scans, the median follow-up time was 19 mo (range, 7–33 mo). Patients with identified primary tumors were managed with a specific regimen, whereas patients with unidentified primary tumors were treated by referring to the guidelines for HNCUP. The progression-free survival rate of patients with identified and unidentified primary tumors was 96% (44/46) and 78% (35/45), respectively. Moreover, the Kaplan–Meier curve (Fig. 3) illustrates that patients with unidentified primary tumors had a significantly worse prognosis than those with identified primary tumors (hazard ratio, 5.77; 95% CI, 1.86–17.94; *P* = 0.0097).

## DISCUSSION

To our knowledge, this study is the largest prospective study investigating the performance of ^68^Ga-FAPI PET/CT compared with ^18^F-FDG PET/CT in detecting primary tumors in patients with HNCUP. Our results demonstrated that ^68^Ga-FAPI PET/CT presented significantly higher diagnostic accuracy (51% vs. 19%, *P* < 0.001) and radioactive uptake (SUV_max_, 6.11 ± 4.30 and 3.16 ± 5.11, *P* < 0.001; TLR, 10.85 ± 6.81 and 1.45 ± 2.31, *P* < 0.001) in localizing primary tumors than did ^18^F-FDG PET/CT. Meanwhile, ^68^Ga-FAPI PET/CT led to treatment changes in 22 of 91 (24%) patients compared with ^18^F-FDG PET/CT. Furthermore, ^68^Ga-FAPI PET/CT outperformed ^18^F-FDG PET/CT in detecting lymph node and bone metastases in terms of TLR.

Our data also highlighted that the prognosis of patients was significantly improved by identifying the primary tumors (*P* = 0.0097; Fig. 3). Recent studies have shown that the 5-y overall survival of HNCUP patients is still dismal, approximately 55% (21). In addition, Faisal et al. (22) reported that the late detection of primary tumors in HNCUP patients after treatment may lead to significantly worse 5-y overall survival than that of HNCUP patients in whom primary tumors remain unidentified. Thus, accurate diagnosis of the primary tumor before treatment is crucial for patients with HNCUP.

Because the oropharynx is the most common primary location for HNCUP malignancies, diagnostic tonsillectomy is recommended for patients with metastatic squamous cell carcinoma of the neck when the primary tumor cannot be identified by noninvasive diagnostic methods, according to American Society of Clinical Oncology guidelines (4). However, only 18%–47% of patients with HNCUP could benefit from diagnostic tonsillectomy (23–25). ^18^F-FDG PET/CT, as a noninvasive, whole-body, and tumor-specific imaging modality, has been widely accepted for locating and clinically staging primary tumors before treatment (26). Significant visual differences between the tumor and the background on PET/CT images could effectively guide the biopsy of suspected malignant lesions. However, physiologic or inflammatory ^18^F-FDG uptake in the head and neck may hide small primary tumors, especially those in the oropharynx (27). In our current study, ^18^F-FDG PET/CT missed 16 of 21 primary tumors in the oropharynx, which is consistent with the research of Pencharz et al. (27). Surprisingly, ^68^Ga-FAPI PET/CT detected all 21 primary tumors in the oropharynx, with significantly higher uptake than in the contralateral normal oropharynx (Supplemental Fig. 3). In line with our research, Serfling et al. (18) demonstrated higher ^68^Ga-FAPI than ^18^F-FDG avidity within malignant primary tumors in the Waldeyer tonsillar ring. Furthermore, Mona et al. (28) reported stronger FAP expression in malignant oropharyngeal lesions than in nonmalignant tissue and a strong correlation between the uptake of ^68^Ga-FAPI and the FAP immunohistochemistry score. Therefore, our research further demonstrates that ^68^Ga-FAPI PET/CT could avoid invasive diagnostic tonsillectomy in patients with HNCUP.

In the current study, although ^68^Ga-FAPI PET/CT detected all 46 primary tumors, which were confirmed pathologically or clinically, the overall sensitivity and accuracy seemed unsatisfactory (51% for each characteristic). This may be because the other 45 patients presented with inconspicuous primary tumors on imaging and endoscopy. Even so, ^68^Ga-FAPI PET/CT could identify small, mucous, and adenoid carcinomas, which always presented non–^18^F-FDG avidity (Table 1) (10). Kratochwil et al. (29) and Chen et al. (13) demonstrated that ^68^Ga-FAPI was a broad-spectrum tumor imaging probe that outperformed ^18^F-FDG in delineating the primary and metastatic lesions in patients with head and neck cancers, gynecologic malignancies, and gastrointestinal cancers, among others. Furthermore, Chen et al. (13) demonstrated the superiority of ^68^Ga-FAPI PET/CT to ^18^F-FDG PET/CT in detecting very small (diameter < 1.0 cm) malignant lesions. In line with the results of the Chen et al. (13) study, our results indicate the potential value of ^68^Ga-FAPI PET/CT in delineating small primary lesions (Supplemental Fig. 2).

The accurate detection of metastatic lesions is helpful in making treatment-related decisions, especially for HNCUP patients. Previous studies (30,31) have shown the apparent advantage of ^68^Ga-FAPI PET/CT over ^18^F-FDG PET/CT in detecting regional and distant metastatic lesions. Wang et al. (30) reported that ^68^Ga-FAPI PET/CT outperformed ^18^F-FDG PET/CT in the detection of advanced lung cancer metastases to the brain, lymph nodes, bone, and pleura. In another study (31), ^68^Ga-FAPI PET/CT revealed significantly higher accuracy than ^18^F-FDG PET/CT in the evaluation of the N0 neck status of oral squamous cell carcinoma patients (100% vs. 29%), which could overcome the potential false-positivity of ^18^F-FDG PET/CT. In our current study, dual-tracer PET/CT detected the same number of metastatic lesions (121 lymph node metastases and 15 bone metastases). In addition, more favorable uptake of ^68^Ga-FAPI than ^18^F-FDG in terms of TLR was presented by both lymph node metastases (18.65 ± 10.50 and 5.64 ± 2.81, *P* < 0.001) and bone metastases (21.99 ± 9.70 and 4.94 ± 2.90, *P* < 0.001), which indicates that FAP-targeted radioligand therapy may exert a strong antitumor effect with little damage to organs at risk (32).

The major limitation of this study is the absence of a histopathologic analysis of tissue samples from primary and metastatic lesions for FAP expression. Because some lesions were examined by fine-needle aspiration, there were no remaining specimens for further immunohistochemistry. Another limitation is that this trial was performed at a single center. In the future, a multicenter trial needs to be performed to verify our results.

## CONCLUSION

Our study demonstrated that ^68^Ga-FAPI PET/CT has higher sensitivity, positive predictive value, and accuracy in locating the primary tumors in HNCUP patients than does ^18^F-FDG PET/CT, which indicates that ^68^Ga-FAPI PET/CT could serve as a sensitive, reliable, and reproducible indicator of primary tumors in HNCUP patients.

## DISCLOSURE

This work was funded by the Special Clinical Research Project of Health Industry of the Shanghai Municipal Health Commission (grant 20224Y0238), the National Natural Science Foundation of China (grant 82272035), and the Talent development project of the Shanghai Public Health System Construction Three-Year Action Plan (GWVI-11.2-YQ49). Data generated or analyzed during this study are available from the corresponding author by request. No other potential conflict of interest relevant to this article was reported.

## Notes

KEY POINTS **QUESTION:** What is the diagnostic accuracy of ^68^Ga-FAPI PET/CT imaging for localization of the primary tumor of HNCUP? **PERTINENT FINDINGS:** In this prospective comparative imaging trial of 91 patients, ^68^Ga-FAPI PET/CT detected more primary lesions than did ^18^F-FDG PET/CT (46 vs. 17 primary tumors) and outperformed ^18^F-FDG PET/CT in sensitivity, positive predictive value, and accuracy for locating the primary tumor (51% vs. 25%, 98% vs. 43%, and 51% vs. 19%, respectively). **IMPLICATIONS FOR PATIENT CARE:** ^68^Ga-FAPI PET/CT outperforms ^18^F-FDG PET/CT in detecting primary lesions and could serve as a sensitive, reliable, and reproducible imaging modality for HNCUP patients.
